# Characterization of *Haemophilus influenzae* Strains with Non-Enzymatic Resistance to β-Lactam Antibiotics Caused by Mutations in the PBP3 Gene in the Czech Republic in 2010–2018

**DOI:** 10.3390/life11111260

**Published:** 2021-11-18

**Authors:** Vladislav Jakubu, Lucia Malisova, Martin Musilek, Katarina Pomorska, Helena Zemlickova

**Affiliations:** 1 National Reference Laboratory for Antibiotics, Centre for Epidemiology and Microbiology, National Institute of Public Health, 10000 Prague, Czech Republic; vladislav.jakubu@szu.cz (V.J.); lucia.malisova@szu.cz (L.M.); katarina.pomorska@szu.cz (K.P.); 2 Department of Clinical Microbiology, Faculty of Medicine and University Hospital, Charles University, 53002 Hradec Kralove, Czech Republic; 3 Department of Microbiology, 3rd Faculty of Medicine, Kralovske Vinohrady University Hospital and National Institute of Public Health, Charles University, 10000 Prague, Czech Republic; 4 National Reference Laboratory for Meningococcal Infections, Centre for Epidemiology and Microbiology, National Institute of Public Health, 10000 Prague, Czech Republic; martin.musilek@szu.cz

**Keywords:** *Haemophilus influenzae*, *ftsI*, β-lactam antibiotics, non-enzymatic resistance

## Abstract

The surveillance data on antibiotic resistance of *Haemophilus influenzae* have shown that strains with non-enzymatic resistance to β-lactam antibiotics have been on the rise in the Czech Republic over the last decade. This type of resistance is more difficult to detect than β-lactamase production. Analysis of 228 *H. influenzae* strains revealed that isolates with non-enzymatic resistance to β-lactams due to mutations in the *ftsI* gene could be reliably demonstrated by single run testing of susceptibility to amoxicillin/clavulanic acid (sensitivity of detection is 84.6%), cefuroxime (92.6%), ampicillin and penicillin (both 95.7%). Thirty-seven different amino acid substitution combinations were detected in the PBP3 protein at 23 positions (V329I, D350N, S357N, A368T, M377I, S385T, A388V, L389F, P393L, A437S, I449V, G490E, I491V, R501L, A502S, A502T, A502V, V511A, R517H, I519L, N526K, A530S, and T532S). The most common combination (35%) of amino acid substitutions was the combination D350N, M377I, A502V, N526K. Epidemiological typing does not indicate a clonal spread of a particular MLST type. Altogether there has been detected 74 STs. The most prevalent ST 1034 was associated mainly with a combination D350N, M377I, A502V, N526K. Clonal analysis revealed six clonal complexes (CCs) with the founder found, eight CCs without founder and 33 singletons.

## 1. Introduction

*Haemophilus influenzae* is part of commensal microflora of the nasopharynx in humans but at the same time remains one of the main causes of a wide range of infections from uncomplicated infections of the upper respiratory tract (sinusitis, otitis media, etc.) to invasive life-threatening conditions (meningitis, epiglottitis, bacteremia, and pneumonia [1].

Aminopenicillins are the agents of choice for non-invasive *Haemophilus* infections [2]. The first strains of *H. influenzae* resistant to ampicillin were reported in the 1970s in Europe and the United States [3]. The mechanism of resistance was found to be a production of a plasmid-mediated β-lactamase, which is inhibited by clavulanic acid or sulbactam. The strains are termed β-lactamase-positive ampicillin-resistant isolates, BLPAR. Two different types of β-lactamases, TEM-1 and ROB-1, have been reported in *H. influenzae* [3,4].

Ampicillin resistance in *H. influenzae* due to a mechanism of resistance other than the production of β-lactamase was reported in the 1980s [5]. It is a structural alteration of the penicillin-binding protein (PBP3) encoded by the *ftsI* gene. Isolates showing this type of resistance are known as β-lactamase negative ampicillin-resistant (BLNAR) or rPBP3, and, unlike in β-lactamase-producing strains, their reduced susceptibility to aminopenicillins is not affected by β-lactamase inhibitors. The product of the *ftsI* gene is transpeptidase FtsI, which is involved in peptidoglycan synthesis. Mutations in the *ftsI* gene result in amino acid (AA) substitutions in the transpeptidase region of the PBP3 protein. The consequence of these AA substitutions is reduced affinity of aminopenicillins and cephalosporins for protein binding. The affinity of β-lactams is affected only by mutations (AA substitutions) near the functionally necessary highly conserved amino acid motifs at positions 327 (S327-T-V motif; serine-threonine-valine), 379 (S379-S-N motif; serine-serine-asparagine), and 512 (K512-T-G motif; lysine-threonine-glycine) of the PBP3 protein. Both mechanisms of resistance, enzymatic and non-enzymatic, could be combined with each other [5,6,7,8]. Other mechanisms of resistance have been described, such as the overexpression of the AcrAB efflux pump caused by mutations in the *acrR* regulatory gene [9].

*H. influenzae* strains with rPBP3 are classified into three basic genotypic groups according to the presence of specific AA substitutions [5]. The genotype with altered PBP3 conferring resistance is referred to as rPBP3. sPBP3 genotype includes neither substitution N526K nor R517H and contains only mutations in the *ftsI* gene that do not result in β-lactam resistance. It is the position of the mutations that determines to which β-lactams and to what extent the strain will be resistant. The most commonly found mutations are the substitution of asparagine for lysine at position 526 (N526K) and the substitution of arginine for histidine at position 517 (R517H). Group I contains the R517H substitution, and Group II carries the N526K substitution. Group III additionally contains mutations in the vicinity of the S379S-S-N381 motif, in particular the S385T. Group II is further divided into other subcategories according to the presence of other specific mutations. Groups I and II are characterized by low resistance to β-lactams, while Group III isolates are highly resistant to β-lactams, including cephalosporins of the second generation (cefuroxime) and even of the third generation (cefotaxime, ceftriaxone), and carbapenems [5,6,7]. β-lactamase production remains the predominant mechanism of resistance in Europe. Although the proportion of BLPAR isolates has been relatively stable over the past decades, rPBP3 is an increasing concern in Europe, as incidences have risen, with group II rPBP3 isolates being particularly common [10,11,12,13].

To test the possibilities for phenotypic detection of rPBP3 strains in routine practice using the disc diffusion method with selected β-lactams, microbiological laboratories in the Czech Republic were invited to send *H. influenzae* strains with suspected non-enzymatic resistance to the National reference laboratory for antibiotics (NRL for ATB). These isolates were sequenced to find out individual amino acid substitutions and whether the spread of rPBP3 strains in the Czech Republic is clonal.

## 2. Results

### 2.1. Phenotypic Detection

A total of 228 *H. influenzae* isolates were examined at NRL for ATB from 2010 to 2018. Thirty-two (14.0%) strains were positive for bla_TEM-1_ and one (0.4%) for the bla_ROB-1_ gene. The ROB-1 β-lactamase producing strain was susceptible to penicillin, and the detection of β-lactamase by the nitrocefin method was negative. The isolates have been classified into all four PBP3 groups. Fifteen strains (6.6%) belonged to rPBP3 Group I and 175 strains (76.8%) to rPBP3 Group II. Most strains in these groups were resistant, but there were also strains susceptible to different β-lactams (Table 1). For further calculations, minimal inhibitory concentration (MIC), not disk diffusion method, is taken into account for ampicillin. All strains (*n* = 26, 11.4%) from rPBP3 Group III were resistant to ampicillin (MIC range 4–16 mg/L), and 88.5% (*n* = 23) of strains showed resistance to cefotaxime. The MIC range of cefotaxime in rPBP3 Group III was 0.125–2 mg/L. Only 12 (5.3%) isolates were assigned to the sPBP3 Group. However, resistance to cefuroxime was found in three strains in this group. Individual combinations of AA substitutions in strains of rPBP3 Groups I and II showed different levels of resistance to ampicillin, which often fluctuated from susceptible to resistant even within the given combination, see Table 1. The MIC_50_ value exceeded the breakpoint of ampicillin (>1 mg/L) in 17 combinations, and the MIC_90_ breakpoint was exceeded in 19 out of 22 combinations found in β-lactamase negative strains of rPBP3 Groups I and II. The sensitivity of detection of mutations in the *ftsI* gene for individual antibiotic discs ranged from 84.6% (amoxicillin/clavulanic acid) to 92.6% (cefuroxime), 95.7% (ampicillin) and 95.7% (penicillin). As many as 187 of 188 (99.5%) β-lactamase negative rPBP strains showed resistance to at least one of the tested antibiotics. The results of MIC of other tested antibiotics (ciprofloxacin, tetracycline, chloramphenicol, trimethoprim-sulfamethoxazole) are listed in Supplementary Table S1.

### 2.2. PCR and Mutation of the ftsI Gene

Comparative analysis of 228 isolates revealed 23 different (AA) substitutions: V329I, D350N, S357N, A368T, M377I, S385T, A388V, L389F, P393L, A437S, I449V, G490E, I491V, R501L, A502S, A502T, A502V, V511A, R517H, I519L, N526K, A530S, and T532S. A total of 37 possible combinations of these AA substitutions were found in the study (Table 1). The AA substitutions occurred predominantly in combinations of two to eight AA substitutions (*n* = 213), with only 15 strains containing five different single-AA substitutions. Three strains with a single AA substitution, N526K, belonged to Group II, and 12 strains containing different substitutions (A502S, V511A, D350N, and A388V) belonged to the sPBP3 genotype. The most common was the combination of four substitutions (D350N, M377I, A502V, and N526K), which was carried by 35% of strains (*n* = 80). This combination was consistently prevalent across time periods, 2010–2014: 33.3%, 2015: 31.7%, 2016: 33.8%, 2017: 39.4%, and 2018: 25%. The N526K substitution occurred in combination (25x) or alone in 184 strains (80.7%). The second basic substitution, R517H, was found in six combinations (33 strains, 14.5%). The N526K and R517H substitutions were not found together in any combination. When substitutions occurred in combinations, the substitution N526K or R517H was always present. In addition to the basic mutation N526K or R517H, all of Group III strains (*n* = 26) also possessed mutations S385T, M377I and others (Table 1).

### 2.3. MLST, CC

**Figure 1:**
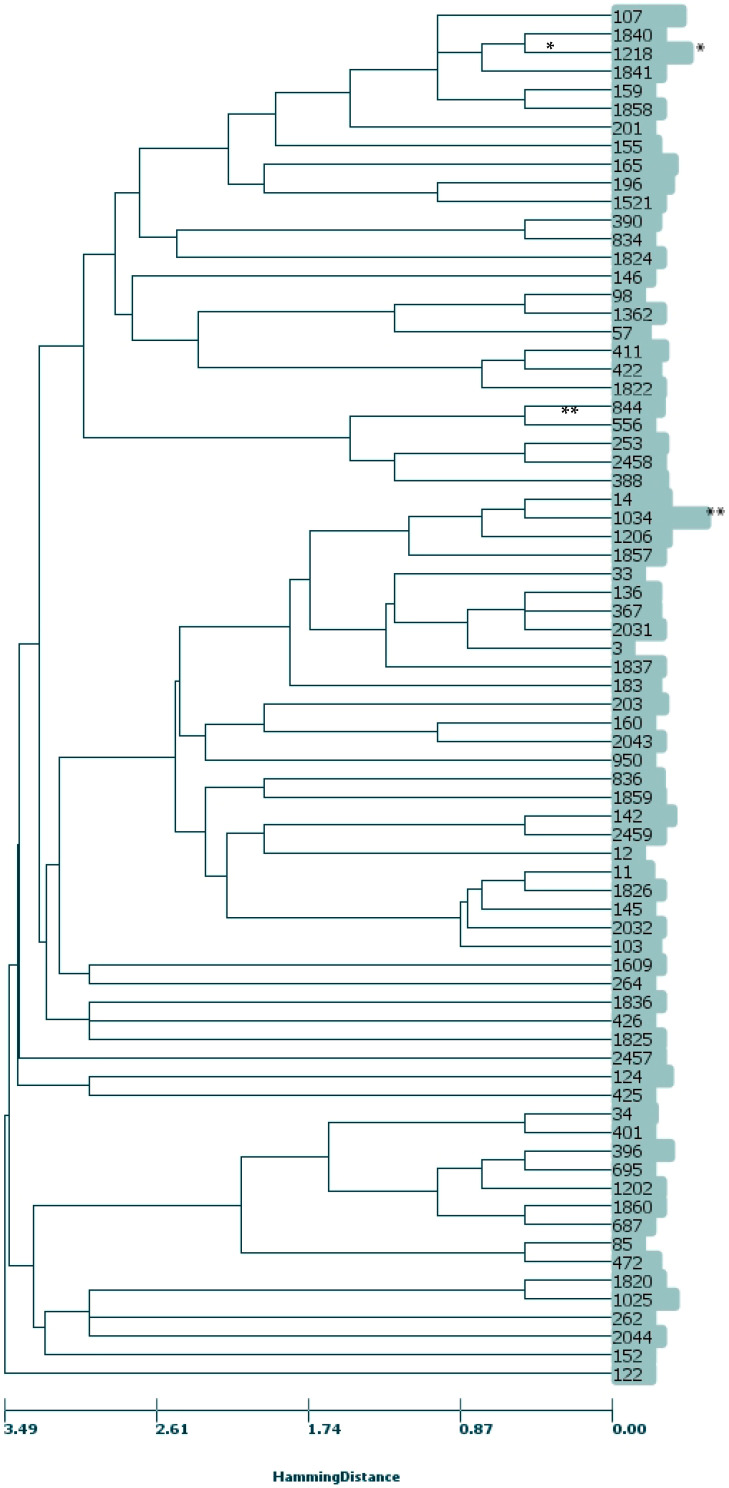
The result of MLST-Hierarchical Clustering analysis (UPGMA method, Scale distance: 150; scale height: 16; cut off threshold: 3.49) of 228 strains of *Haemophilus influenzae*. Numbers are representing the names of STs. All figures were generated (computed and visualized) by PHYLOViZ 2.0 (www.phyloviz.net, accessed on 10 December 2020). The phylogenetic distance of individual STs indicates a wide origin and little interrelationship between the rPBP3 Group I, Group II, Group III and sPBP strains. * ST 1218 (*n* = 13, 5.7%) was the most frequent ST with R517H substitution (combination A502V, R517H, *n* = 11). ** ST 1034-the most represented ST in the whole set (*n* = 49, 21.5%) of strains, of which 48 had the most common combination of AA substitutions in the study: D350N, M377I, A502V, and N526K.

A total of 74 different STs were determined in the collection of 228 strains, including 15 new STs: ST 1820, ST 1822, ST 1824, ST 1825, ST 1826, ST 1836, ST 1837, ST 1840, ST 1841, ST2 032, ST 2043, ST 2044, ST2 457, ST 2458, and ST2459, see Table 1. Six new alleles (*aptG* (allele 138, ST 2458), *frdB* (197, ST 2044), *mdh* (285, ST 1825; 303, ST 2043; and 325, ST 2457), and *pgi* (272, ST 1826)) were discovered. The nine new STs were new combinations of the already known alleles. In four STs, the number of strains reached 10 or more: ST 1034 (*n* = 49, 21.5%), ST 103 (*n* = 17, 7.5%), ST 14 (*n* = 13, 5.7%), and ST 1218 (*n* = 13, 5.7%). ST 1034 has been dominant since 2014 (31.6%), with an oscillation of incidence in the following years between 15–28%. The mutual phylogenetic distance between individual STs is shown in Figure 1. In the strains (*n* = 80) with the most frequent combination of mutations (D350N, M377I, A502V, and N526K), 11 different STs were identified, of which ST 1034 dominated (*n* = 47; 58.8%). Clonal analysis computed as a single locus variant of alleles (eBURST algorithm) revealed six clonal complexes (CCs) with the founder found (a total of 113 strains), eight CCs without founder (containing only 2 ST; *n* = 36) and 33 singletons (*n* = 79). The CCs with the founder found were: CC 11 (*n* = 8, ST 11, ST 103, ST 145, ST 1826, and ST 2032), CC 14 (*n* = 64, ST 14, ST 1034, and ST 1206), CC 367 (*n* = 6, ST 3, ST 136, ST 367, and ST 2031), CC 396 (*n* = 11, ST 396, ST 687, ST 1202, and ST 1860), CC 422 (*n* = 7, ST 411, ST 422, and ST1822), and CC 1218 (*n* = 17, ST 159, ST 1218, ST 1840, ST 1841, and ST 1858).

## 3. Discussion

The surveillance data on antibiotic resistance in *H. influenzae* causing respiratory tract infections (sinusitis, acute otitis media, and community-acquired pneumonia) collected annually by the NRL for ATB have shown a growing trend (Supplementary Figure S1) in *H. influenzae* strains with non-enzymatic resistance to β-lactam antibiotics in the Czech Republic [14]. Since 2010, there has been an overall increase in resistance to ampicillin in *H. influenzae*, from 8.6% in 2010 to 22.1% in 2019. Within the same period, the proportion of rPBP3 strains rose from 0.8% to 7.7%. Thus, while the rate of BLPAR is almost constant, the proportion of rPBP3 strains has increased 10-fold since 2010 [14]. In the first years of surveillance, the occurrence of rPBP3 strains was rare; since 2014, the prevalence has increased to a maximum in 2017 (71 strains).

Our project is the first comprehensive work in the Czech Republic to deal with the occurrence of rPBP *H. influenzae*. It is focused on the description of phenotypic detection of rPBP3 strains, their clonal distribution, as well as a detailed analysis of AA substitutions in the PBP3 gene. Since 2015, occasional strains resistant to cefotaxime have also been confirmed in the NRL for ATB [15].

The sensitivity of detection of non-enzymatic resistance by individual β-lactam antibiotics was determined in 228 strains. The highest sensitivity of detection of rPBP3 strains was shown by a screening test with 1 U penicillin (95.7%). This is in accordance with EUCAST and, for example, the results of Aguirre’s results [16]. The same sensitivity (95.7%) was shown by a test with ampicillin (2 µg). Screening with penicillin and ampicillin in a single run may be recommended for strains with mutations in PBP3, reaching a combined sensitivity of 98.9%. Only two (0.9%) β-lactamase negative rPBP strains (AA combination A502V, R517H) were susceptible to both penicillin and ampicillin. Differences in ampicillin susceptibility testing results between the MIC and disk diffusion methods were noted, as confirmed by the results of other studies [17,18].

Comparative analysis revealed 37 combinations of 23 different AA substitutions in the PBP3 protein in our collection of 228 strains. These numbers are higher than in similar studies [16,19,20], probably due to a larger number of strains or a longer observed period of time. The N526K substitution, the most commonly found substitution in the world [5,10,11,16], was also the most frequent substitution in our study.

The most common combination of substitutions in the tested collection was D350N, M377I, A502V, and N526K, which was carried by 35% (*n* = 80) of strains. The above-mentioned combination was very common in some countries [16,19]. The largest number of AA substitutions in a single strain was eight, and they occurred in five cefotaxime resistant strains. Due to a large number of AA substitutions and their combinations, it is not possible to determine the specific contribution of each substitution to the β-lactam resistance. As an example, the V329I is the unique substitution, which is the only one of the 23 substitutions to be found in the region of the highly conserved AA motif (S327-T-V). This substitution conferring resistance to cefotaxime has only been reported once [21]. In our collection, it is present in a single cefotaxime resistant strain with the combination of AA substitutions V329I, D350N, S357N, M377I, S385T, L389F, and N526K. However, only the S385T together with M377I substitutions are sufficient for a cefotaxime MIC of around 1 mg/L [22,23]. As can be seen, even mutations directly affecting the conserved motif do not confer extra high resistance to cefotaxime.

The second most common substitution is D350N, which was observed in 19 combinations and also alone (151 strains in total, 66.2%). This substitution is most common, for example, in Australia [13]. Of the eight strains carrying the D350N substitution alone, five were susceptible to all tested β-lactams. Its contribution to the resistance is therefore questionable. Very similar, A388V substitution was detected in a single strain, which produced β-lactamase but was susceptible to both amoxicillin/clavulanate and cefuroxime. Some authors [20,24] have reported that these substitutions, along with A368T and R510L, have no effect on resistance. The A502 position is the most variable considering AA in the PBP3 protein in our study strains. There were found three different substitutions at this position, A502S, A502T, and A502V. Substitutions at position A502 are the third most common (found in 16 combinations or alone, for a total of 134 strains, 58.8%). According to Skaare [10] and Tristam [24], A502V replacement confers low-level resistance to β-lactams. However, substitutions at this position also do not always seem to confer resistance. This can be inferred from the fact that one strain with a single AA substitution (A502S) was susceptible to all β-lactams.

In our study set, there were 26 strains of rPBP Group III, with M377I, S385T, and N526K or R517H substitutions found in all these strains. The substitutions S385T and N256K or R517H are necessary for resistance to the 3rd generation cephalosporins [5,22,23]. To one detected combination of substitutions, this assumption did not apply: three of the five strains with the D350N, S357N, M377I, S385T, R517H, and T532S substitution combinations were susceptible to the third generation cephalosporins (MIC cefotaxime of 0.125 mg/L). In contrast, cefotaxime resistant strains were found in Group II in the combinations D350N, G490E, A502V, N526K, A530S (one strain with β-lactamase production) and M377I, I449V, N526K (two out of five strains with the same combination of AA mutations without β-lactamase production). Since the 1990s, cefotaxime-resistant rPBP isolates have been associated with infections, especially in certain Asian regions [23,25,26]. In European countries, the occurrence was very rare until 2008, and the first clonal spread was described from Norway [27].

The MLST method was used to determine the epidemiological relationship of the strains. The subsequent clonal phylogenetic analyses showed heterogeneity of the rPBP3 *H. influenzae* population. Only 20% of the strains sent from 19 laboratories from all administrative regions of the Czech Republic were clonal. The group of 49 strains carried the combination of AA substitutions D350N, M377I, A502V, N526K and belongs to ST 1034. A similarly fragmented population has been reported, for example, from Norway, Spain, Italy, and Japan [28,29,30,31,32]. Even when comparing the phylogenetic distance between the rPBP3 individual groups, MLST analysis showed a low relatedness between Groups I, II, and III of the rPBP3 strains, represented by ST 1218 and ST 1034. ST 1218 predominated in Group I (*n* = 12/15) and is absent in groups Groups II and III. ST 1034 was prevalent in the Czech Republic only, and ST 1218 was found to be dominant in Japan [32]. Neither the strains isolated from blood (*n* = 25) nor the β-lactamase producing strains (*n* = 33) formed homogeneous groups, belonging to 17 and 19 different STs, respectively. The cefotaxime resistant strains (*n* = 26) were also diverse and comprised of 15 different STs.

Our study has some limitations. Clonal experiments to confirm the contribution of individual AA substitutions to β-lactam resistance have not been performed and may be the subject of further studies. According to Thegerström [33], even artificially cloned mutations, other than those detected in this collection, reduced susceptibility to aminopenicillins. This suggests that the range of mutations leading to resistance is wide, and new AA substitutions may continue to occur. Experiments to detect other known mechanisms of resistance that could affect the susceptibility to β-lactams have not been performed. The question is how many associated mechanisms of resistance, such as the overexpression of the AcrAB efflux pump [9], and to what extent may have been involved in the resulting susceptibility. This could explain the wide range of susceptibility values found within a single AA combination. Our unpublished data show that mutations in the AcrR repressor of the AcrAB efflux pump can lead to resistance even in the absence of AA substitutions in the *ftsI* gene.

## 4. Materials and Methods

### 4.1. Strains

From 2010 to 2018, 228 strains of *H. influenzae* with non-enzymatic resistance to β-lactams were sent to the NRL for ATB (National Institute of Public Health, Prague, Czech Republic) from 39 laboratories from all 14 administrative regions of the Czech Republic (2010–2014: *n* = 33 strains, 2015: *n* = 41, 2016: *n* = 68, 2017: *n* = 71, and 2018: *n* = 16). Strains were isolated from sputum (*n* = 104), nose swab (*n* = 31), blood (*n* = 23), throat swab (*n* = 21), ear (*n* = 17), cerebrospinal fluid (*n* = 8), puncture fluid (*n* = 6), eye swab (*n* = 3), vagina swab (*n* = 2), and the origin of 13 strains was unknown. When selecting strains, the laboratories followed the EUCAST recommendations [34]: β-lactamase negative strains (with an inhibition zone of <12 mm around the penicillin disc), β-lactamase positive strains (resistant to antibiotics effective against β-lactamase, i.e., amoxicillin/clavulanic acid, cefuroxime) were sent to the NRL for ATB. The obtained strains were inoculated on Levinthal agar (Oxoid, the Czech Republic) and incubated overnight at 35 ± 1 °C and 5% CO_2_. The identification was performed using MALDI-TOF MS (Microflex, Bruker Daltonics, Germany).

### 4.2. Susceptibility Testing to β-Lactams

The NRL for ATB tested the susceptibility to penicillin (1 U), cefuroxime (30 µg), ampicillin (2 µg), and amoxicillin/clavulanic acid (2/1 µg) using the disk diffusion method. The minimal inhibitory concentration (MIC) of cefotaxime and ampicillin was determined by the broth microdilution method. Both the disk diffusion method and the broth microdilution method were performed according to the EUCAST methodology [34]. The interpretation of susceptibility testing results was conducted as recommended by the EUCAST guidelines, version 10.0. Breakpoints for resistance were set as follows: penicillin (<12 mm), ampicillin (<18 mm), ampicillin MIC > 1 mg/L, amoxicillin/clavulanic acid (<15 mm), and cefuroxime (<25 mm). *H. influenzae* strains ATCC 49766 and ATCC 49247 were used as quality controls. β-lactamase production was examined by the nitrocefin method [35]. β-lactamase negative strains were defined as rPBP3 with resistance to at least one of the β-lactam antibiotics. β-lactamase positive strains were defined as rPBP3 when they were resistant to amoxicillin/clavulanic acid or cefuroxime.

### 4.3. PCR Detection of β-Lactamases

Verification of β-lactamase production was performed by PCR detection of bla_TEM-1_ and bla_ROB-1_ [36]. *H. influenzae* strains CIP 107112 and CIP 104278 were used as positive controls for the detection of the bla_TEM-1_ and bla_ROB-1_ genes, respectively.

### 4.4. Detection of Mutations in the ftsI Gene and Comparative Analysis

All strains were sequenced in the 977–1597 bp region of the *ftsI* gene (621 bp fragment) to verify the presence of mutations in the PBP3 transpeptidase region according to the protocol from PubMLST.org [37]. The nucleotide sequence was converted into an amino acid (AA) sequence in Bionumerics 7.6.2 (Applied Maths, Ghent, East Flanders, Belgium) and compared with the AA sequence (positions 326–532) of the non-mutated reference strain *H. influenzae* ATCC 51907 (GenBank accession number L42023) [38].

### 4.5. Multi-Locus Sequence Typing (MLST) and Clonal Analysis

Multi-locus sequence typing (MLST) was performed in all 228 strains. Briefly, approximately 450-bp internal fragments of seven housekeeping genes (*adk*, *atpG*, *frdB*, *fucK*, *mdh*, *pgi*, *and recA*) were amplified by PCR using primers and methods previously described [39].

Allele and sequence type (ST) numbers were assigned using Bionumerics 7.6.2 (Applied Maths, Ghent, East Flanders, Belgium) and the free access website (Public databases for molecular typing and microbial genome diversity, https://pubmlst.org/organisms/haemophilus-influenzae, accessed on 25 May 2020).

Grouping population into clonal complexes was performed by the goeBURST algorithm [40] generated by PHYLOViZ (https://www.phyloviz.net, accessed on 20 May 2020). The default program settings were used (hierarchical clustering-hamming distance; unweighted pair group method with arithmetic mean; cut-off threshold 3.48). A clonal complex was defined as a group of STs that have at least six alleles in common with another member of the defined group [39].

## 5. Conclusions

In conclusion, the combinations of AA substitutions and heterogeneous populations of rPBP strains found are consistent with the results reported by others. Strains with N526K and strains with R517H were found, and the most common combination of AA substitutions is D350N, M377I, A502V, and N526K. In the Czech Republic, the proportion of rPBP strains among ampicillin-resistant strains is constantly increasing [14]. The large number of cefotaxime-resistant strains is also alarming. It is therefore highly appropriate to continue antimicrobial resistance surveillance at the national level and to teach clinical laboratories to reliably recognize rPBP strains in a clinical specimen. Improved detection of these strains will reduce their spread. For maximum reliability of phenotypic detection of all known types of *ftsI* gene mutations, all four β-lactam antibiotics should be tested in a single run by disk diffusion method.

## Figures and Tables

**Table 1:** Table presents the combinations of substitutions (grouped into resistance groups) detected in this study, range of susceptibility to antibiotics, and results of MLST typing of 228 human clinical isolates of *Haemophilus influenzae*.

		β-Lactamase Negative								β-Lactamase Positive				
			Range of Measured Values						MLST		Range of Measured Values			MLST
PBP3 Groups	AA Substitutions and Their Combinations	N	MIC AMP ^c^ (mg/L)	MIC CTX (mg/L)	PEN (mm)	AMC (mm)	CRX (mm)	AMP MIC_50_/MIC_90_ (mg/L)	ST/CC * ^a^	N	MIC CTX (mg/L)	AMC (mm)	CRX (mm)	ST/CC * ^a^
sPBP3 (*n* = 12)	A502S	1	1	0.06	12	15	26	1/1	1218/1218					
	V511A									1	0.06	11	23	426
	D350N	6	0.25–1	0.016–0.03	12–16	17–21	26–28	0.25/0.5	124 (5), 145/11	3	0.016	15–16	21–29	388 (3)
	A388V									1	0.03	17	27	401
rPBP3 Group I (*n* = 15)	A502V, R517H	13	1–4	0.03–0.125	6–19	12–19	14–27	2/4	1218/1218 (11), **1840**/1218, **1841**/1218	2 ^b^	0.016–0.06	13–19	23–31	146, 1218/1218
rPBP3 Group II (*n* = 175)	A368T, A502T, N526K	2	1–4	0.06	6–11	17	20–23	1/4	950, **1825**					
	A502T, N526K	1	2	0.125	6	13	21	2	12					
	A502V, N526K	5	1–2	0.03–0.06	6–11	11–15	21–25	2/2	142 (2), 411/422 (3)	2	0.03–0.06	10–14	21–23	34, 411/422
	D350N, A437S, A502V, N526K	6	1–4	0.03–0.06	6–11	9–16	15–23	2/4	196 (5), **1837**	2	0.03–0.06	10–13	17–23	836 (2)
	D350N, A437S, G490E, N526K, A530S	1	4	0.06	6	7	21	1	390					
	D350N, A502T, N526K	8	1–4	0.06–0.125	6–11	10–14	6–21	2/4	183 (2), 1362, **1820**, 1025 (4)	1	0.06	9	19	1860/396
	D350N, A502V, N526K	1	4	0.06	10	14	22	4	122					
	D350N, G490E, A502V, N526K	1	2	0.03	9	15	21	2	**2457**					
	D350N, G490E, A502V, N526K, A530S									1	0.25	6	12	836
	D350N, G490E, N526K, A530S	18	0.25–4	0.008–0.06	6–11	10–18	17–25	2/4	107 (14), 159/1218, 201, 203, 425					
	D350N, I449V, N526K	1	1	0.03	12	16	24	1	152					
	D350N, M377I, A502V, N526K	74	0.25–16	0.016–0.125	6–11	6–20	11–29	2/8	14/14 (13), 98, 124, 136/367 (2), 145/11, 165 (6), 203 (3), 262, 367/367 (2), 422/422, 1034/14 (47), 1206/14 (2), 1218/1218	6	0.03–0.125	6–14	15–23	165 (5), 1206/14
	D350N, M377I, G490E, A502V, N526K	6	1	0.03–0.06	6–11	10–14	18–21	1/1	33, 556, 834, 844 (3)					
	D350N, M377I, I491V, A502V, N526K	1	4	0.125	6	10	18	4	1034/14					
	D350N, S357N, A502V, N526K	1	2	0.125	9	12	17	2	**1822**/422					
	G490E, N526K, A530S	1	2	0.125	9	12	19	2	1858/1218					
	G490E; A502V; N526K	1	2	0.06	10	14	17	2	85					
	I449V, N526K	17	1–4	0.03–0.06	6–10	9–15	15–27	2/4	57, 253 (4), 390, 396/396 (7), 687/396, 695/396, 1202/396, **2458**					
	M377I, A502V, N526K									3	0.03–0.06	11–15	19–20	165 (3)
	M377I, I449V, N526K	5	1–4	0.06–0.25	6	6–14	12–19	4/4	11/11 (3), **1826**/11, **2032**/11					
	N526K	3	0.5–4	0.03–0.06	8–11	13–17	19–27	1/4	34 (3),					
	N526K, A530S	5	1–4	0.06–0.125	6–11	12–17	17–25	2/4	**2459**, 142 (4)					
	R501L, N526K	2	1	0.06	6–8	12–13	19–24	1/1	196 (2)					
rPBP3 Group III (*n* = 26)	D350N, M377I, S385T, L389F, I519L, N526K	1	4	0.5	6	6	6	4	**2044**					
	D350N, S357N, M377I, S385T, L389F, G490E, N526K, A530S	2	4–8	0.5	6	6–12	6–12	4/8	107, 1859	2	0.5	6–16	8–16	107 (2)
	D350N, S357N, M377I, S385T, L389F, N526K	2	4–16	1	6	6	6	4/16	472 (2)					
	D350N, S357N, M377I, S385T, L389F, P393L, R517H, T532S									1	1	6	6	388
	D350N, S357N, M377I, S385T, L389F, R517H									1	1	12	16	422/422
	D350N, S357N, M377I, S385T, R517H, T532S	5	1–8	0.125–1	6–11	8–15	6–21	4/8	57, 155 (2), 1857, 2031/367					
	D350N, S357N, M377I, S385T, L389F, R517H, T532S	3	16	1–2	6	6–11	6–17	16/16	142, 264, **2043**	6	1–2	6–12	6	3/367, 103/11 (2), 160, 1521, **1824**
	M377I, S385T, L389F, R517H	1	8	1	6	6	6	8/8	142	1	1	6	6	**1836**
	V329I, D350N, S357N, M377I, S385T, L389F, N526K	1	8	2	6	13	15	8/8	1609					

N, number of strains; ST, sequence type; CC, clonal complex; MIC, minimal inhibitory concentration; AMP, ampicillin; AMC, amoxicillin/clavulanic acid; CRX, cefuroxime; CTX, cefotaxime; PEN, penicillin; ^a^—number of strains of the ST in brackets (only if the number is greater than 1); ^b^—one strain contained beta-lactamase ROB1; ^c^—for results of the disk diffusion method, see Supplementary Table S1. *—CC is given only for complexes with a found founder; ST without the indicated CC belongs to singletons or without founder; **Bold**—newly described ST; Italics—resistance values.

## Data Availability

The data presented in this study are available on request from the corresponding author.

## Supplementary Materials

The following are available online at https://www.mdpi.com/article/10.3390/life11111260/s1, Supplementary Table S1: Result of individual strains; Supplementary Figure S1: Trend of resistance to β-lactam antibiotics in H. influenzae in years 2010–2019 and proportion of enzymatic and non-enzymatic mechanism of resistance in the Czech Republic.
