# Supplementary material for: Characterization of Haemophilus influenzae Strains with Non-Enzymatic Resistance to β-Lactam Antibiotics Caused by Mutations in the PBP3 Gene in the Czech Republic in 2010–2018
Source: Life (Basel). 2021 Nov 18;11(11):1260. doi: 10.3390/life11111260 (PMC8624647; doi:10.3390/life11111260)
Supplement: Supplementary file 1 [file life-11-01260-s001.zip › life-1423560-SI/supplementary/Figure S1 Trend of resistance to a┬-lactam antibiotics in H. influenzae in years 2010¿C2019 and proportion of enzymatic and non-enzymatic mechanism of resistance in the Czec.pdf]

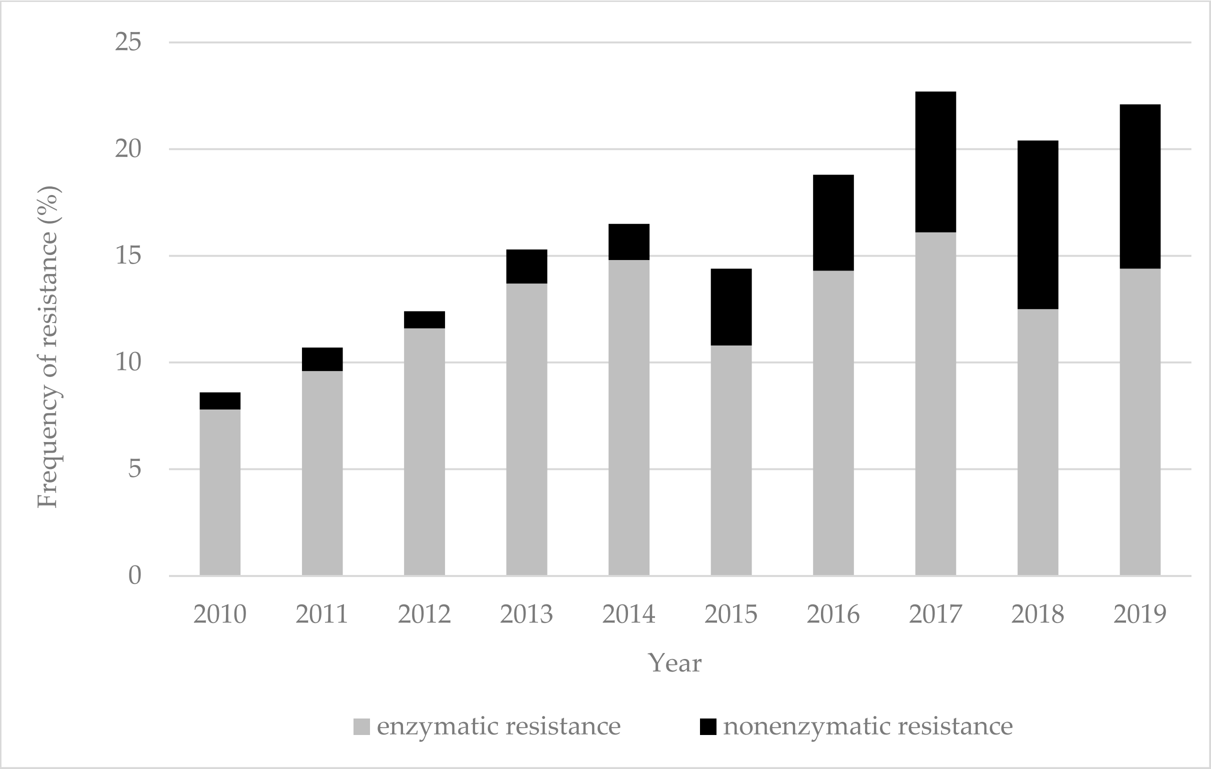

Figure S1. Trend of resistance to β-lactam antibiotics in *H. influenzae* in years 2010 - 2019 and proportion of enzymatic and non-enzymatic mechanism of resistance in the Czech Republic. The graph is based on the surveillance data on resistance to ampicillin and amoxicillin / clavulanic acid of *H. influenzae* causing respiratory tract infections (http://www.szu.cz/respiracni-studie-atb-rezistence). The data (https://apps.szu.cz/rp/respiracni_patogeny.php) are collected annually by the National reference laboratory for antibiotics, National Institute of Public Health, Prague, the Czech Republic.
